# Hospital Construction

**Published:** 1899-04-22

**Authors:** 


### The Institutional Workshop.

### HOSPITAL CONSTRUCTION.

### TREATMENT OF HOSPITAL SEWAGE.

The Infectious Diseases Hospital at Manston has adopted a system of drainage which, in practice, has proved so satisfactory that an explanation may be useful. The drainage is discharged into "Dibdin's tanks," and this method of sewage disposal is still new in connection with hospitals. Our readers are probably aware that the system which is associated with the name of Mr. W. J. Dibdin, lately chemist to the London County Council, is one which has been in use on a portion of the sewage of London, at Barking Creek,* since November, 1896, and has been adopted by the town of Sutton, Surrey, with such success that other places are beginning to make use of the advantages in economy and simplicity which it offers. Briefly, the system, in which more than one worker has had his part, consists in passing the sewage through a layer of coke breeze, charged with the living organisms derived from the sewage itself, and in so regulating the flow of the sewage over these "bacteria beds" that their tiny inhabitants have time to consume, destroy, and convert the whole of the organic matter in the sewage into harmless constituents, which pass out into the effluent without making it in any way injurious.

In the short space at our disposal it is impossible to enter into details as to the manner in which these results are obtained. Those who are interested in the question, as promoters of new hospitals or otherwise, are advised to study Mr. Dibdin's well-known work on "The Purification of Sewage and Water," from which we gather that the main principles to be observed are (1) that the filter must be gradually brought to a state of efficiency by regulated charges of the effluent, which contains the necessary organisms; (2) that the sewage must be allowed to rest in contact with the filter for a period depending on the amount of purification required; (3) that after each period of action the organisms must be supplied with air, which is effected by draining off the effluent from below; and (4) that a base, such as lime, must be present with which the nitric acid resulting from the process can combine.

A filter of this description, properly constructed and regulated, will cost, where the soil is suitable, about £600 per acre, and an acre will purify one million gallons of sewage per day, at an expense which is nominal, compared with most other systems, sometimes weighted with heavy royalties in addition to the actual cost of working. Experiments with infected sewage show that no difficulty arises in its disposal by the same means, the bacteria having no conscientious objections to typhoid fever or other diseases. The system is in use satisfactorily at the Sutton Isolation Hospital on Banstead Downs; and its adoption at Manston is another instance showing that it is likely to prove of advantage to hospitals established in country districts, as well as to local authorities dealing with the sewage of towns.

* See an account of these works in an article on "The Thames and the Sewage," in The Hospital, Nov. 14 and 21, 1896.

### NEW INFIRMARY FOR THE PARISH OF ST. MARY, ISLINGTON, HIGHGATE HILL.

This infirmary, which is erected on the grounds of the late small-pox hospital, Highgate Hill, and, in fact, includes in its administrative department the building of the old hospital, is intended to accommodate 800 patients. The new buildings are arranged on a symmetrical plan, five double pavilions branching out on either side of a main corridor, which runs from west-north-west to east-south-east, or thereabouts. The central pair of these double pavilions are devoted to stores, kitchens, dining-rooms, offices, &c., and are of lower elevation than the others. Owing to the fall of the land the ward blocks facing south contain one storey more than those to the north, there being four storeys on the one side against only three on the other. Each of the main south wards contains 30 beds, and attached to each is a separation ward, which will hold two or three beds. The wards on the north side are not all the same size, the two outer blocks having wards of 28 beds each, while those nearest the kitchen department have wards of only 16 beds. They will, however, have separation wards, and the same kitchen, larder, linen-room, and lavatory accommodation. This is a very good arrangement, the administration of a large hospital being greatly facilitated by there being some variety in the size of the wards.

In regard to the wards themselves it is to be noted that neither the floor space nor the wall space is very liberal, the latter being only just over six feet per bed, while the floor space works out to under 76 feet per bed. The beds are so arranged that there are two between each pair of windows. This is an arrangement which is clearly necessary where the wall space per bed is so small, and we have no doubt that in this case it has been necessity rather than choice which has led to the plan being adopted. We think, however, that hospital architects would do well to reconsider the whole question of windows. It seems quite clear that the desire to interpose a window between each bed and its neighbour has led in many cases to a distribution of window space which is far from beautiful, and most annoying to the patients. How often do we not find it laid down that a patient is not to be placed opposite to a window. But there are many hospitals in which every patient must of necessity be opposite a window, and in which the narrow strip of wall space between the lights affords no rest to the eyes. In this particular some of the workhouse infirmaries are better arranged than many new hospitals. Each large ward is provided with a very good balcony day-room, and these rooms, where they face to the south, ought to be very pleasant and useful adjuncts. Each ward is cut off from the main corridor by a cross-ventilating lobby, and the bath-rooms and lavatories are also cut off from the wards in the same manner. As in all modern poor-law hospitals, there is a complete system of fire-escape galleries. Extra bath-rooms are placed in the corridors so that patients can be bathed before entering the wards. Each double pavilion is provided with a lift, and attached to each pair of wards are separate lavatory arrangements, which, we presume, are intended for the use of the nurses—a very necessary provision where their work is so far from their residence. The old buildings of the small-pox hospital have been rearranged, so as to provide residences for the medical superintendent, assistant medical officers, matron, &c., and a nurses' home for about 90 nurses. Behind this building are placed the washhouses and laundry, and the dead-house and post-mortem rooms. Altogether it is a very satisfactory building, and one well suited for the work for which it is intended. Mr. William Smith, A R.I.B.A., is the architect, and Messrs. Kirk and Randall are the contractors. The estimate for the work was £181,583.

